# Cognitive function in people with and without freezing of gait in Parkinson’s disease

**DOI:** 10.1038/s41531-020-0111-7

**Published:** 2020-05-15

**Authors:** Rosie Morris, Katrijn Smulders, Daniel S. Peterson, Martina Mancini, Patricia Carlson-Kuhta, John G. Nutt, Fay B. Horak

**Affiliations:** 1 Oregon Health & Science University, Portland, OR USA; 2 Northumbria University, Newcastle upon Tyne, UK; 3 Sint Maartenskliniek, Nijmegen, The Netherlands; 4 Arizona State University, Phoenix, AZ USA; 5 Phoenix VA Medical Center, Phoenix, AZ USA; 6 VA Portland Health Care System, Portland, OR USA

**Keywords:** Parkinson's disease, Movement disorders

## Abstract

Freezing of gait (FOG) is common in people with Parkinson’s disease (PD) which is extremely debilitating. One hypothesis for the cause of FOG episodes is impaired cognitive control, however, this is still in debate in the literature. We aimed to assess a comprehensive range of cognitive tests in older adults and people with Parkinson’s with and without FOG and associate FOG severity with cognitive performance. A total of 227 participants took part in the study which included 80 healthy older adults, 81 people with PD who did not have FOG and 66 people with PD and FOG. A comprehensive battery of neuropsychological assessments tested cognitive domains of global cognition, executive function/attention, working memory, and visuospatial function. The severity of FOG was assessed using the new FOG questionnaire and an objective FOG severity score. Cognitive performance was compared between groups using an ANCOVA adjusting for age, gender, years of education and disease severity. Correlations between cognitive performance and FOG severity were analyzed using partial correlations. Cognitive differences were observed between older adults and PD for domains of global cognition, executive function/attention, and working memory. Between those with and without FOG, there were differences for global cognition and executive function/attention, but these differences disappeared when adjusting for covariates. There were no associations between FOG severity and cognitive performance. This study identified no significant difference in cognition between those with and without FOG when adjusting for covariates, particularly disease severity. This may demonstrate that complex rehabilitation programs may be undertaken in those with FOG.

## Introduction

Freezing of gait (FOG) is one of the most problematic motor symptoms in Parkinson’s disease (PD) affecting over 60% of patients with a disease duration of 10 years or more^1^. Furthermore, FOG contributes to an increased risk of falls, reduced quality of life, increased mood disorders and increased caregiver burden^2–4^. Not all patients with PD develop FOG. Incidence increases with disease duration but clinically it is difficult to predict which patients will transition to develop FOG. A number of factors contribute to FOG risk which include age, anxiety, depression, and severity of motor symptoms but the role of cognition in the development of FOG is still debated in the literature^5–8^.

**Fig. 1:**
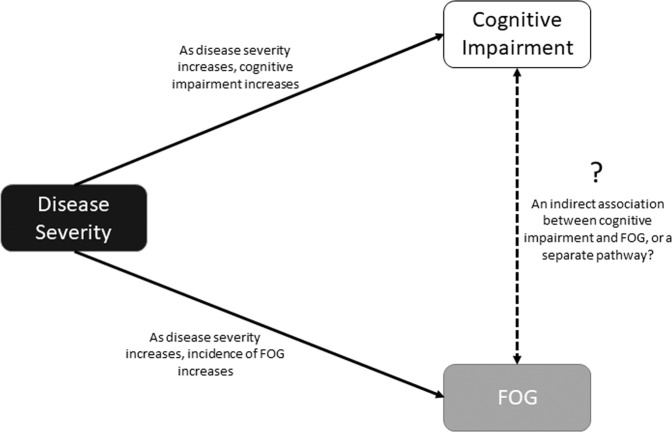
The association between disease severity, cognitive impairment and, freezing of gait (FOG). Disease severity directly impacts on FOG and cognitive impairment. The indirect association between FOG and cognitive impairment remains unknown.

Impaired cognitive function is common in PD, particularly for frontal lobe functions of attention and executive function with difficulties present at disease diagnosis^9^. A number of studies have looked at differences in cognition between those with FOG (FOG+) and without FOG (FOG−) suggesting that domains of executive function, attention, and visuospatial function are worse in FOG+ compared to FOG−^10–14^. However, most studies to date are within small cohorts and do not control for disease severity. The association between cognitive decline and PD severity, and PD severity and FOG is difficult to disentangle (Fig. 1). Therefore, it is critical that disease severity is taken into account when comparing cognitive function in those with and without FOG.

The severity of FOG is assessed in the majority of cases using self-report questionnaires dependent on patient subjective recall^15,16^. To better characterize and quantify FOG, an objective measure may be better suited for assessment across participants and when assessing relationships between FOG and behavioral outcomes. Mancini et al., previously developed an objective outcome measure of FOG that provides a continuous ratio score validated against both the New FOG self-reported questionnaire and expert neurologist evaluation^17,18^.

This study, therefore, aimed to: (i) assess a comprehensive range of cognitive domains in a large cohort of older adults and PD FOG+ and FOG− and (ii) associate cognitive performance with FOG severity using subjective and objective FOG measures. We hypothesize that those with PD will have poorer cognitive function compared to older adults and specific to PD, measures of executive function, attention, and visuospatial ability will be worse in FOG+ compared to FOG−. In addition, PD participants with more severe objective FOG scores will demonstrate poorer attention, executive function and visuospatial abilities compared to those with milder FOG.

## Results

### Demographic and clinical assessments

A total of 227 participants were recruited to the study; 147 of which were diagnosed with idiopathic PD and 80 healthy older adults (OA). Of those with PD, 66 were classified as FOG+ and 81 were classified as FOG−. Demographic and clinical assessments are shown in Table 1. When comparing OA and PD, there were no differences for age or gender but those with PD had a poorer MoCA score (OA; 26.87 ± 2.27, PD; 25.75 ± 3.40, *p* < 0.01). When comparing FOG+ and FOG−, there was no significant difference for age, gender or MoCA (Table 1). However, motor disease severity, as measured by the MDS-UPDRS III, was significantly greater in the FOG+ group (45.92 ± 12.44) compared to the FOG− group (36.20 ± 10.83, *p* < 0.001). The FOG+ group had significantly greater FOG severity, as demonstrated by the FOG ratio score (2.65 ± 6.03) compared to FOG− (0.68 ± 0.76, *p* < 0.01).

**Table 1:** Demographic and clinical characteristics for controls, PD and PD without freezing (FOG−) and PD with freezing (FOG+).

		OA (*n* = 80)	PD (*n* = 147)	PD FOG− (*n* = 81)	PD FOG+ (*n* = 66)	Independent T-test	
						OA vs PD	FOG+ vs FOG−
Age (yrs)		68.21 (8.07)	68.48 (8.06)	68.80 (8.01)	68.08 (8.17)	0.814	0.589
Gender (M/F)^a^		48/32	95/52	50/31	45/21	0.490	0.416
Education (years)		16.61 (1.86)	16.23 (1.90)	16.20 (1.87)	16.26 (1.96)	0.150	0.841
MDS UPDRS-III		N/A	40.56 (12.52)	36.20 (10.83)	45.92 (12.44)	N/A	<0.001
Disease duration (years)		N/A	6.23 (4.93)	4.93 (4.19)	7.83 (5.31)	N/A	<0.001
MoCA		26.87 (2.27)	25.75 (3.40)	26.00 (3.05)	25.45 (3.80)	<0.01	0.342
NFog Score		N/A	N/A	N/A	12.00 (7.02)	N/A	N/A
FOG Ratio Score		0.41 (0.35)	1.55 (4.16)	0.68 (0.76)	2.65 (6.03)	<0.01	<0.01
H&Y^a^	1	N/A	1 (0.7%)	1 (1.2%)	0 (0%)	N/A	<0.01
	2	N/A	117 (79.6%)	72 (88.9%)	45 (68.2%)		
	3	N/A	16 (10.9%)	5 (6.2%)	11 (16.7%)		
	4	N/A	13 (8.8%)	3 (3.7%)	10 (15.2%)		

^a^Chi-squared test.

### Cognitive performance between OA and PD and FOG+ and FOG−

**Fig. 2:**
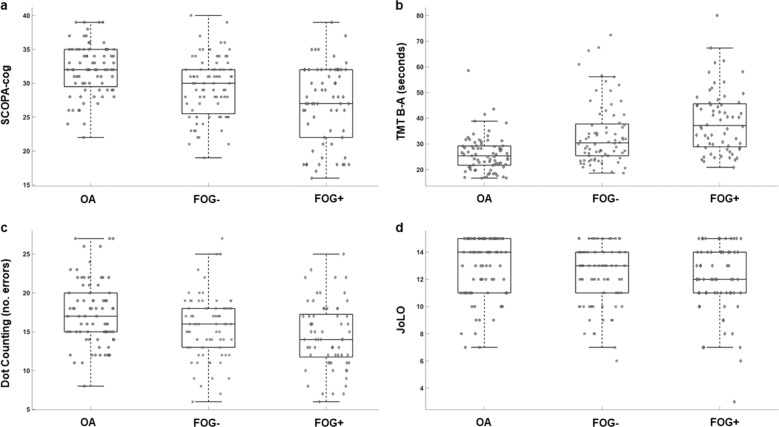
Scatterplots representing cognitive function in older adults (OA), non-freezers (FOG−) and Freezers (FOG+). Cognitive performance for **a** Scopa-cog, **b** Trail making Test B–A, **c** Dot counting and **d** JoLO.

Independent sample *t*-tests without covariates indicated that the PD group had worse performance than the OA group for all cognitive tests except for Go-NoGo (*p* = 0.02) and JoLO (*p* = 0.04), see Table 2. Uncorrected, independent sample *t*-tests also showed worse cognitive performance for the FoG+ compared to FoG− for global cognition (SCOPA-COG, *p* < 0.01) and in the executive function/attention domain for the Flankers test (*p* < 0.01), but no other tests (see Table 2). Box and scatter plot representations of cognitive assessments for OA, FOG−, and FOG+ are shown in Fig. 2.

**Table 2:** ANCOVA for cognitive differences between FOG+ and FOG−.

Cognitive Domain	Control (*n* = 80)	PD (*n* = 147)	PD FOG− (*n* = 81)	PD FOG+ (*n* = 66)	Independent T-test		ANCOVA	
					Control vs PD	FOG− vs FOG+	Control vs PD^a^	FOG− vs FOG+^b^
Global cognition								
Scopa-Cog	32.00 (3.75)	28.26 (5.18)	29.38 (4.38)	26.89 (5.76)	<0.001	<0.01	<0.001	0.016
Executive function/attention								
SRT (m/sec)	315.69 (27.07)	342.23 (52.89)	339.98 (50.56)	345.06 (55.98)	<0.001	0.574	<0.001	0.716
TMT B-A (sec)	11.83 (9.28)	21.45 (30.89)	15.59 (20.92)	28.53 (38.74)	<0.001	0.019	0.031	0.054
Stroop color (sec)	31.62 (6.92)	35.91 (00.04)	34.19 (7.99)	37.98 (11.79)	<0.001	0.028	<0.01	0.235
Stroop interference (sec)	64.85 (16.92)	76.61 (31.78)	72.36 (26.87)	81.69 (36.37)	<0.001	0.087	<0.01	0.113
Flankers	8.73 (0.39)	8.29 (0.67)	8.44 (0.45)	8.10 (0.83)	<0.001	<0.01	<0.001	0.062
Go-NoGo (Accuracy)	74.24 (16.53)	68.37 (19.20)	71.06 (18.51)	64.99 (19.65)	0.02	0.063	0.036	0.199
Working memory								
Dot counting (errors)	17.45 (4.22)	15.12 (4.13)	15.66 (3.97)	14.46 (4.26)	<0.001	0.086	<0.001	0.301
Visuospatial function								
Visuospatial functionJoLO	12.80 (2.24)	12.11 (2.37)	12.21 (2.18)	12.00 (2.59)	0.039	0.608	0.047	0.784

^a^Controlling for age, gender and years of education.

^b^Controlling for age, gender, years of education and MDS-UPDRS III.

Adjusting for age, gender and years of education, resulted in fewer differences between PD and OA groups, with the PD group exhibiting poorer global cognition (SCOPA-COG; *p* < 0.01), poorer executive function/attention (SRT (*p* < 0.01), Stroop color (*p* < 0.01), Stroop Interference (*p* < 0.01) and Flankers (*p* < 0.01) and poorer working memory (Dot counting; *p* < 0.01) (Table 2). When comparing FOG+ and FOG− and adjusting for age, gender, years of education and disease severity (MDS-UPDRS III) there were no significant differences between groups. To further understand the effect of individual covariates, analyses were run controlling for individual covariates (Supplementary Table 1) which revealed the MDS-UPDRS III had the greatest effect on cognitive performance.

### Association between FOG severity and cognitive performance

**Fig. 3:**
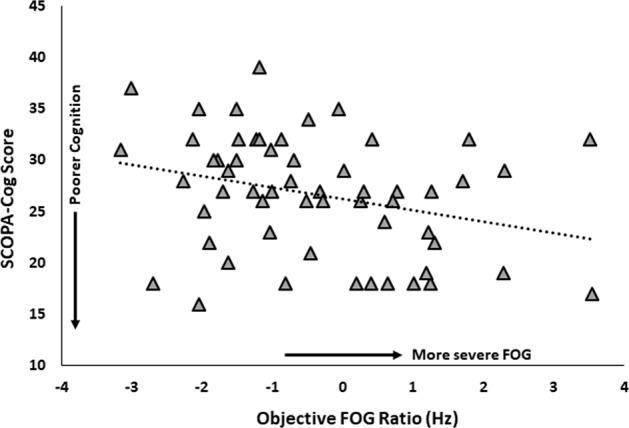
The relationship between objective FOG severity and global cognition assessed by scales for outcomes in Parkinson’s disease-cognition (SCOPA-Cog). Higher FOG ratio denotes more severe FOG, higher SCOPA-Cog score denotes better cognition.

Cognitive performance was not correlated with either self-reported FOG severity or objective FOG severity in any of the groups (Table 3). The objective FOG ratio was weakly correlated with the SCOPA-Cog in FOG+ (*r* = −0.28; *p* = 0.04), suggesting those with poorer global cognition had greater freezing severity, but this did not reach the stringent significance level adjusted for multiple comparisons (Fig. 3).

**Table 3:** Partial correlations between objective FOG ratio and subjective questionnaire with cognitive domains.

	Correlation with FOG Ratio			Correlation with FOG Total Score		
	OA	FOG−	FOG+	OA	FOG−	FOG+
Global Cognition						
Scopa-Cog	−0.217 (0.089)	0.124 (0.325)	−0.280 (0.040)	N/A	N/A	−0.130 (0.350)
Executive function/attention						
Simple reaction time	−0.030 (0.817)	−0.077 (0.541)	0.022 (0.877)	N/A	N/A	0.119 (0.390)
TMT B-A	−0.160 (0.213)	0.129 (0.305)	−0.017 (0.905)	N/A	N/A	0.216 (0.116)
Stroop color	−0.068 (0.600)	−0.115 (0.360)	0.027 (0.849)	N/A	N/A	−0.033 (0.812)
Stroop interference	0.070 (0.586)	0.188 (0.133)	0.050 (0.719)	N/A	N/A	0.222 (0.106)
Flankers test	0.135 (0.296)	0.241 (0.053)	−0.034 (0.806)	N/A	N/A	−0.178 (0.199)
Go-NoGo	0.148 (0.251)	−0.023 (0.853)	−0.151 (0.276)	N/A	N/A	−0.031 (0.823)
Working memory						
Dot counting	0.067 (0.607)	0.116 (0.359)	0.252 (0.066)	N/A	N/A	−0.110 (0.428)
Visuospatial function						
JoLO	0.005 (0.971)	−0.111 (0.379)	0.028 (0.842)	N/A	N/A	0.088 (0.525)

Presented are Pearson’s correlation coefficients and *p*-values (in brackets). Controlling for age, gender and years of education.

## Discussion

This large study compared a comprehensive range of neuropsychological assessments in older adults and people with Parkinson’s disease, with and without freezing of gait. Although the FOG+ group showed poorer executive and global cognitive function compared to the FOG− group, this difference was not significant when controlling for covariates, particularly disease severity. As supported by previous work, this suggests that cognitive deficits and FOG incidence may occur concomitantly as PD progresses. In addition, we observed no associations between FOG severity and cognitive impairment when using subjective or objective measures of FOG severity, suggesting that severity of FOG may not relate to severity of cognitive impairment.

Patients with PD demonstrate cognitive deficits, even in early disease^9^, and our analysis demonstrates established cognitive impairments in this cohort of PD compared to healthy older adults. In our analysis, we identified significant differences between FOG+ and FOG− when we did not adjust for covariates; however, when controlling for factors reflecting more advanced disease progression, the differences were no longer significant. Further analysis revealed that adjusting for disease severity eliminated differences between FOG+ and FOG−, with these findings corroborated by the weak association between FOG severity and cognitive task performance. Incidence of cognitive impairment and FOG are more likely as disease progresses^1^ and therefore FOG may occur via two parallel process of increasing motor disease severity advancing cognitive impairment (Fig. 1). One theory underlying FOG episodes stipulates functional decoupling between neural networks, identifying that the level of functional connectivity between cognitive and motor areas may trigger FOG episodes^19^. Therefore, connectivity may become further impaired as disease progresses leading to FOG episodes, independent of level of cognitive function. Furthermore, we previously identified a loss of white matter fibers in an executive inhibition pathway between the right SMA and right STN in PD with FOG^20^. Therefore, cognition may play a role in some instances of FOG but this may occur indirectly via disease severity dependent on other factors.

A number of studies have also demonstrated worse cognition in FOG across a number of domains, such as executive function and attention^10,12,13,21^. However, the majority of studies contain small sample sizes and as a result, often do not control for confounders which may affect cognition e.g. disease severity and age. Unlike our study, the majority of studies assess cognition ‘on’ dopaminergic medication^5,10,11,13,14,21,22^ whereas our patients were assessed in the ‘off’ state. Medication status in PD not only influences motor symptoms, but can also affect non-motor symptoms, including cognition both in a positive and negative manner^23,24^. The role of dopamine on cognition is complex, evidence suggests that dopamine improves a number of cognitive processes but in addition, overload of the dopaminergic system may result in reduced cognitive performance for other cognitive processes^25–29^. Furthermore, cognitive training for people with FOG demonstrated significant improvement in FOG episodes when in the ‘on’ state but not the ‘off’ state^30^. This suggests an interplay between dopamine and cognition that underpins FOG. Importantly, we compared our PD cohort to age-matched older adults and demonstrated a similar pattern of cognitive impairment to other studies in the literature. However, future studies should assess cognition in FOG+ and FOG− in large cohorts both on and off medication to better understand dopaminergic influence on cognitive pathways in those who experience FOG.

Severity of FOG has previously been associated with severity of cognitive deficit i.e. the more severe the FOG, the greater the cognitive deficit^10,12,14^. However, we did not find any associations between the severity of freezing and cognitive function either with self-report or objective measures of FOG severity. In order to improve the accuracy of FOG severity, we used an objective FOG ratio to provide scores for OA, FOG− and FOG+. In our FOG+ group, there was a stronger association between FOG severity ratio score and global cognition and working memory compared to the FOG questionnaire score. It is possible that the objective FOG ratio score provides a more sensitive metric of FOG severity and may provide a useful outcome measure for future studies.

Our study had a number of strengths including a large cohort of PD allowing for large and near equal size groups for FOG+ and FOG−. Furthermore, we assessed a comprehensive range of executive function and attention cognitive assessments, the domain most noted to be different between FOG+ and FOG− in previous work. However, our study also had a number of limitations. First, our cognitive battery had a smaller number of tests assessing memory and visuospatial function and therefore our results may be biased towards frontal executive function and attention. Second, although not necessarily a limitation, our participants were all assessed ‘off’ medication and therefore our results may be difficult to compare to other studies. Furthermore, we compared our PD cohort ‘off’ medication to a group of age-matched older adults and identified cognitive impairments were similar to other studies in the literature.

Overall in this large study, we found no statistical differences in cognitive outcomes between people with PD who do and do not freeze when taking disease severity into account. Our findings may have future implications for rehabilitation of FOG, however, findings will need to be validated in a future cohort.

## Methods

### Participants

Participants diagnosed with idiopathic PD and healthy age-matched older adults (OA) were recruited to the study at Oregon Health and Science University and the VA Portland Healthcare system. All PD participants were screened by a movement disorders specialist at either Oregon Health and Science University or the VA Portland Healthcare System. OA and PD participants were included in the study if they were aged between 50 and 90 years old. People with PD were included in the study if they (i) were diagnosed with idiopathic PD according to UK Brain Bank Criteria^31^ and (ii) had no other neurological disorder other than PD. Participants were excluded if they had cognitive difficulties so that they could not follow instructions. Informed consent was gained from all participants All subjects provided informed consent approved by the joint Institutional Review Boards at Oregon Health & Science University (4131) and the VA Portland Health Care System (8979).

### FOG Classification

PD participants were classified as FOG+ if: (i) they answered yes to the first question on the New Freezing of Gait Questionnaire (NFOG-Q)^15^ (Did you experience any freezing of gait episodes within the last month?) after seeing an accompanying video showing examples of FOG episodes, and/or (ii) an episode of freezing was observed during a laboratory assessment. All participants were assessed in the practical ‘Off’ medication state, with medication withdrawn a minimum of 12 h prior to assessments.

### Clinical assessments

Age, gender, years of education, and global cognitive function via the Montreal Cognitive Assessment (MoCA) score^32^ were recorded for all participants. To assess PD severity, disease duration in years was recorded and motor severity was assessed using part III of the Movement Disorders Society Unified Parkinson’s disease rating scale (MDS-UPDRS-III)^33^ and Hoehn and Yahr score (H & Y)^34^. The severity of FOG was recorded subjectively using the NFOG-Q and objectively using the FOG ratio score^17,18^. The FOG ratio score was calculated from anterior-posterior accelerations from wearable sensors on both ankles whilst participants turned clockwise and counterclockwise 360 degrees for 1 minute. The ratio of power at the freezing frequency band (3–7 Hz) over a walking frequency band (0.5–2 Hz) was calculated for the turning trial.

### Cognitive assessments

Four domains of cognition were assessed: global cognition, executive function/attention, working memory and visuospatial. *Global cognition* was assessed using the Scales for Outcomes in Parkinson’s disease-cognition (SCOPA-COG) which is comprised of ten items assessing attention, working memory, executive function, and visuospatial abilities^35^. *Executive function and attention* were assessed by tests of simple reaction time (SRT), the time to complete the Trail Making Test (TMT) Part B minus Part A, the time to complete the Stroop color condition, the time to complete the Stroop interference condition, the Flankers test, and the Go-NoGo test. *Working memory* was assessed using the Dot Counting test from the NIH examiner battery. Finally, *visuospatial ability* was assessed using the Benton’s Judgement of line orientation (JoLO).

### Statistical analysis

Differences in demographical characteristics were compared between older adults and PD and FOG+ and FOG− using independent samples *t*-tests and chi-square tests where appropriate. Differences in cognitive performance were compared between: (i) PD and OA and (ii) FOG− and FOG+. Comparisons were analyzed first without covariates using independent samples *t*-test. Second, ANCOVA’s were used to detect differences in cognitive performance whilst adjusting for covariates. Covariates of age, gender, years of education and disease severity using the MDS-UPDRS III were selected due to their association with cognitive performance.

To determine associations between FOG severity and both self-report and objective measures of FOG, partial correlations were used controlling for age, gender and years of education. To control for multiple comparisons, statistical significance was set at α < 0.01 throughout the analysis.

The data that support the findings of this study are available from the corresponding author upon reasonable request.

### Reporting summary

Further information on research design is available in the Nature Research Reporting Summary linked to this article.

## Supplementary information

Supplementary Table 1

Reporting Summary

## Data Availability

The data that support the findings of this study are available from the corresponding author upon reasonable request.
