# Supplementary material for: Cognitive function in people with and without freezing of gait in Parkinson’s disease
Source: NPJ Parkinsons Dis. 2020 May 15;6:9. doi: 10.1038/s41531-020-0111-7 (PMC7228938; doi:10.1038/s41531-020-0111-7)
Supplement: Supplementary file 1 — Supplementary Table 1 [file 41531_2020_111_MOESM1_ESM.pdf]

		Model 1	Model 2	Model 3	Model 4	Model 5
		**Age**	**Gender**	**Years of Education**	**MDS-UPDRS III**	**Age, Gender, Education, MDS-UPDRS III**
**Global cognition**	*Scopa-Cog*	11.96 (.001)	7.96 (.005)	8.37 (.004)	2.4 (.124)	5.91 (.016)
**Executive Function/ Attention**	*SRT (m/sec)*	.326 (.569)	.230 (.632)	.262 (.610)	.140 (.708)	.133 (.716)
	TMT B-A (sec)	7.13 (.009)	5.23 (.024)	5.10 (.025)	3.46 (.065)	3.78 (.054)
	Stroop color (sec)	7.97 (.005)	4.53 (.035)	4.29 (.040)	.449 (.504)	1.42 (.235)
	Stroop interference (sec)	5.56 (.020)	2.68 (.104)	2.51 (.116)	.592 (.443)	2.54 (.113)
	Flankers	11.79 (.001)	9.01 (.003)	9.32 (.003)	2.05 (.154)	3.54 (.062)
	Go-NoGo (Accuracy)	3.98 (.048)	2.96 (.087)	2.92 (.089)	1.20 (.276)	1.67 (.199)
**Working memory**	*Dot counting (errors)*	3.78 (.054)	2.29 (.132)	2.76 (.099)	.591 (.444)	1.08 (.301)
**Visuospatial function**	*JoLO*	.288 (.592)	.547 (.461)	.364 (.547)	.000 (.988)	.076 (.784)

Supplementary Table 1. ANCOVA for differences in cognition between FOG+ and FOG- whilst adjusting for covaraites. Model 1, adjusting for age; Model 2, adjusting for gender; Model 3, adjusting for Years of Education; Model 4, adjusting for MDS-UPDRS III (disease severity); Model 5, adjusting for all covariates.
